# "*I never had the money for blood testing" *– Caretakers' experiences of care-seeking for fatal childhood fevers in rural Uganda – a mixed methods study

**DOI:** 10.1186/1472-698X-8-12

**Published:** 2008-12-02

**Authors:** Helena Hildenwall, Göran Tomson, Judith Kaija, George Pariyo, Stefan Peterson

**Affiliations:** 1 Division of International Health (IHCAR), Karolinska Institutet, Stockholm, Sweden; 2 Medical Management Center (MMC), Karolinska Institutet, Stockholm, Sweden; 3 Iganga/Mayuge Demographic Surveillance Site, Uganda; 4 School of Public Health, Makerere University, Kampala, Uganda; 5 International Maternal and Child Health (IMCH), Uppsala University, Sweden

## Abstract

**Background:**

The main killer diseases of children all manifest as acute febrile illness, yet are curable with timely and adequate management. To avoid a fatal outcome, three essential steps must be completed: caretakers must recognize illness, decide to seek care and reach an appropriate source of care, and then receive appropriate treatment. In a fatal outcome some or all of these steps have failed and it remains to be elucidated to what extent these fatal outcomes are caused by local disease perceptions, inappropriate care-seeking or inadequate resources in the family or health system. This study explores caretakers' experiences of care-seeking for childhood febrile illness with fatal outcome in rural Uganda to elucidate the most influential barriers to adequate care.

**Methods:**

A mixed methods approach using structured Verbal/Social autopsy interviews and in-depth interviews was employed with 26 caretakers living in Iganga/Mayuge Demographic Surveillance Site who had lost a child 1–59 months old due to acute febrile illness between March and June 2006. In-depth interviews were analysed using content analysis with deductive category application.

**Results:**

Final categories of barriers to care were: 1) "Illness interpretation barriers" involving children who received delayed or inappropriate care due to caretakers' labelling of the illness, 2) "Barriers to seeking care" with gender roles and household financial constraints hindering adequate care and 3) "Barriers to receiving adequate treatment" revealing discontents with providers and possible deficiencies in quality of care. Resource constraints were identified as the underlying theme for adequate management, both at individual and at health system levels.

**Conclusion:**

The management of severely ill children in this rural setting has several shortcomings. However, the majority of children were seen by an allopathic health care provider during the final illness. Improvements of basic health care for children suffering from acute febrile illness are likely to contribute to a substantial reduction of fatal outcomes. Health care providers at all levels and private as well as public should receive training, support, equipment and supplies to enable basic health care for children suffering from common illnesses.

## Background

Every year, six million children between one month and five years of age die from causes that could be prevented with existing interventions [1]. The majority of these deaths are due to only three diseases: pneumonia, malaria and diarrhoea. Fatal outcomes could be prevented through various existing interventions, but poor care-seeking practices in combination with weak health systems sustain this unacceptably high death toll [2-4].

Care-seeking is a complex process influenced by caretakers' individual circumstances and community health beliefs [5]. Some illness episodes may be interpreted as best suited for traditional care. Observations from eastern Uganda found the emic [6] illness concept "enhonhi" (literally translated "bird disease") to involve symptoms of all three major childhood killers [7], yet the stated treatment preference was herbs. Caretakers may also abstain from seeking care if they fail to recognize symptoms or do not consider them dangerous [5]. Moreover, one disease may be misinterpreted for another; especially where health information has focused on some illness(es) while giving less attention to others. In Uganda, "omusudha" (hot body) is used for any childhood fever [8] and is frequently treated with antimalarial drugs [9]. This may delay treatment for other febrile illnesses, in particular pneumonia since symptoms often overlap with those of malaria [10].

Once a caretaker has recognized illness and decided to seek care, household responsibilities and long distances to health-units may still delay care-seeking [11]. When ultimately reaching a provider, the quality of care received might not be adequate. Hospital care for severely ill children may involve treatment delays, insufficient knowledge among staff, and the need for patients to buy their own drugs [12]. Case management practices are often not in line with national or international guidelines and drug dosage errors are not uncommon [13]. Further health system deficiencies include limited human resources, drug supplies and service management capacities [14]. Despite this documentation of healthcare provision and system deficiencies, little attention has been paid to understanding how users view the quality of care provided and received [15].

To reduce pneumonia mortality, three crucial steps in management have been suggested by UNICEF: "recognize", "seek" and "treat" [16]. These steps are equally important for malaria and diarrhoea. Many child deaths could be averted if timely recognition of symptoms was followed by prompt care-seeking at a place where accurate diagnosis would lead to the administration of the right drug(s) in correct doses. We know that care-seeking delays contribute to child deaths in eastern Uganda [17], but not how caretakers' experience the care-seeking process or their perceptions of how different barriers have contributed to the fatal outcome. The extent to which fatal febrile illnesses are caused by local disease perceptions, inappropriate care-seeking or inadequate resources in the family or health system remains to be elucidated. This study, therefore, aims to explore caretakers' experiences of care-seeking for childhood febrile illness with fatal outcome in order to elucidate the most influential barriers to receiving adequate care.

## Methods

### Study Site

Iganga/Mayuge Demographic Surveillance Site (DSS) is situated 120 kilometers east of Kampala. It includes 65 villages and 67,000 individuals, of whom 12,500 are children under five years. The main ethnic group is Basoga who speak Lusoga. The main economic activity in the region is subsistence farming. The Basoga have a tradition of polygamy, with many men have several wives. Malaria is holoendemic and the region's under-five mortality rate is 116/1000 [18]. Health facilities within the Demographic Surveillance Area (DSA) include Iganga District Hospital with 117 beds, 8 public health centres, 3 NGO clinics, some 122 drug shops and private clinics and numerous traditional healers. Care in public health centres should be free of charge as per government policy. The national treatment recommendations at the time of the study were trimethoprim/sulfamethoxazole for non-severe pneumonia and chloramphenicol for severe cases. Recommendations for non-severe malaria were arthemether-lumefantrine (Coartem^®^). Severe malaria should be treated with quinine and diarrhea with Oral Rehydration Solution and zinc supplementation.

### Data Collection

We used mixed qualitative and quantitative methods [19] with concurrent data collection. Deaths of children 1–59 months old were identified through the regular DSS system. After a mourning period of 4–6 weeks, caretakers were approached and asked to consent to a verbal autopsy interview, collecting information on symptoms that preceded the death. Iganga/Mayuge DSS uses the InDepth Network verbal autopsy (VA) [20] which has been locally adapted and merged with a social autopsy (SA) tool [21] to collect information on the type and timing of care preceding the death.

If caretakers reported that the child had had fever prior to the death, the VA/SA interview was complemented with an open-ended interview about the time preceding the death that included prompts for illness terminology, decision-making processes, and experiences of healthcare sought. Data was collected at the home of the interviewee by two non-medical female interviewers who are native to the area. Interviewers received extensive training in performance of VA/SA interviews and were trained on eliciting in-depth interviews in three piloting sessions.

Interviews were done in Lusoga at the caretakers' home. One or more caretakers could attend the interview but there was one main respondent. The structured VA/SA interviews took 40–60 minutes to complete and the in-depth interview an additional 15–30 minutes. In-depth interviews were tape recorded, transcribed and translated into English by a non-medical Lusoga speaker. The interview probing-list was drafted by the first author and revised after training, piloting and discussion with the two interviewers and co-authors.

### Sample size and data analysis

Interviews were conducted with 26 caretakers living in the DSA who lost a child aged 1–59 months between March and June 2006 due to acute febrile illness. The mixed methods analysis began by analyzing data within each method. The quantitative analysis of VA/SA included descriptive and non-parametric statistics of symptoms observed during the illness, reported timing of home treatment and care-seeking outside the home. Due to the frequent overlap between symptoms of pneumonia and malaria [10] and low positive predictive values of diagnosis from verbal autopsies [22], we chose to study Acute Febrile Illness with and without the key pneumonia symptoms difficult and rapid breathing (DRB). Households were classified as most poor, very poor, poor, less poor and least poor using DSS data of principal component analysis based on household ownership of assets [23].

Qualitative interviews were analyzed using manifest content analysis [24] with deductive category application [25], using the UNICEF-steps "recognize", "seek" and "treat" as sensitizing concepts. This was done by sorting emerging codes into preliminary categories related to recognition of illness, care-seeking and treatment. Several sub-categories emerged during analysis, which lead to modification of the final categories. HH, SP and GT read and reviewed transcripts independently to get an overall understanding of the material. The qualitative software Nvivo^® ^was used to code and group codes into categories. Names and symptoms of local illness concepts in the area obtained from previous work [7] were used to compare agreement between generally stated preferences and action taken in an actual case (Table 1).

**Table 1:** Agreement between symptoms and treatments for different illnesses given in Focus Group Discussions with mothers in other study [7] and action reported in in depth interviews

**Results from Hildenwall et al 2007**			**Results from this study**	
**Local illness concept**	**Explanation/symptoms**	**Treatment preference from Focus Group Discussions (FGDs)**	**Treatment given in accordance with results from FGDs**	**Number ***(some caretakers gave more than one illness name*)

Omusudha	Hot body	Herbs, panadol, antimalarial drugs	Yes	8

Okuwamu omusayi	Lack of blood, pale palms	Blood transfusion	Yes	5

Lukunsense	Measles; fever, vomiting, diarrhoea, rash, coated eyes	Herbs	No, went to hospital for care.	3

Lwenyanja	Diarrhea, fever, desquamation of the skin around the private parts	Traditional care. Sometimes in combination with hospital care	Yes	3

Enhonhi	Cold hands and feet with warm trunk, yellowish diarrhoea, tightness in the chest with difficult breathing.	Herbs	Yes, but went to hospital after giving herbs at home.	2

Lubyamira	Difficult breathing	Hospital care/home care until severe illness	Yes, child put on drugs at home but quickly taken to hospital	1

Okwesika	Convulsions	Traditional care	Yes	1

Ebiino	False teeth; vomiting, diarrhea, high temperature and does not breath well	Traditional care	No, went to hospital for care.	1

Others	Missing information	Missing information	Different management	6

Simple matrices were constructed were quantitative and qualitative data were combined to display experience of the care-seeking process depending on symptoms and type of encountered providers. Children were divided into two groups depending on presence or absence of difficult or rapid breathing (DRB) to reflect treatment of possible pneumonia.

### Ethical Considerations

Informed consent was obtained from all individuals participating in the interviews. Efforts were made to respect the sensitive situation of the bereaved caretakers through empathic approaches and training of interviewers [26]. The study was approved by the Institutional Review Board at Makerere University School of Public Health, Uganda National Council of Science and Technology (HS-32).

## Results

In most interviews, the mother of the deceased was the main respondent. All deceased children were under 2 years of age and there were more boys (n = 16) than girls (n = 10). VA/SA interviews revealed that after fever (n = 26), diarrhoea was the most commonly reported symptom (n = 17), followed by rapid breathing (n = 13) and difficult breathing (n = 12). When combined, 16 caretakers reported recognition of difficult or rapid breathing (DRB). Ten children reportedly presented with chest-indrawing during the illness. One child seemingly suffered from hydrocephalus. Almost half of the children had died at home (n = 11). However, all but three had been in contact with an allopathic healthcare provider during the illness, either private (n = 3), public (n = 12) or both (n = 8). Three children had been taken to a traditional care provider. The illness episodes varied in length from one day to two months. Nine households belonged to the poorest quintile, six to the very poor, six to the poor, four to the less poor and one household belonged to the least poor household (p < 0.05).

From in-depth interviews, "omusudha" (hot body), "okuwamu omusayi" (lack of blood) and "lukunsense" (measles) emerged as the terms most commonly used by caretakers to label the illness (Table 1). Some caretakers also reported "okwesika" (convulsions) or one of the local illness concepts "lwenyanja", "ebiino" and "enhonhi" (Table 1).

The final categories of barriers to adequate care from qualitative analysis were "Illness interpretation barriers" e.g., when interpretation of illness may have caused delayed treatment/care-seeking, "Barriers to seeking care", e.g., financial constraints for seeking care and "Barriers to receiving adequate treatment" e.g., reported provider limitations like long waiting times and lack of drugs. Household and health system resource constraints were identified as a theme running through all categories.

### Illness interpretation barriers

Three children reportedly suffered from "lwenyanja", a traditional illness concept believed to be caused by spirits, manifesting with desquamation of the skin (Table 1). Two of these children first received allopathic care and were then brought to an herbalist who offered herbal bathing in at late stage of illness. One child with "okwesika" had also been treated by a traditional healer since the condition was believed to have been caused by spirits. Two caretakers reported their children had been ill from a disease explained as "enhonhi"; both were initially treated at home and died on their way to hospital.

*"As you know the Basogas' traditional belief that the child has enhonhi, one buys some tablets and *[initiate treatment at home and]*if there isn't any change, that is when medical care is sought in hospital so the truth can be found out." *24 year old mother of deceased 10 month old girl, very poor socioeconomic quintile

Five of the eight caretakers who reported their children had suffered from "omusudha" had also recognized difficult/rapid breathing during the illness. Two of these children received antibiotics while the others reportedly received antimalarials and antipyretics only. One caretaker reported the child had suffered from "lubyamira" (the literal translation of pneumonia), saying this was information received from hospital records.

### Barriers to seeking care

For some children, care-seeking seemed to have been delayed due to the absence of the father, who was described as the person to make the ultimate decision of whether to seek care outside the home. Three mothers gave reports of having to find the father before any decision involving money could be made.

*"The child was admitted but I told the doctor to first discharge us to go and inform my husband. However, we didn't find my husband at his work station in town. So we had to wait until evening to go to the clinic*." 18 year old mother of deceased 8 month old boy, most poor socioeconomic quintile

Neighbours, friends and relatives also influenced the care-seeking, for example by giving suggestions as to what type of illness the child suffered from.

*"We were told by a number of people that the child was sick due to traditional beliefs and were advised to visit a traditional healer." *25 year old mother of deceased 18 month old boy, less poor socioeconomic quintile

According to VA/SA interviews, most children (21/26) were taken to more than one healthcare provider. Care-seeking usually started by visiting a nearby provider (public health centre n = 10 and private clinics n = 10) that could be reached on foot or bicycle. Proximity was the most frequently reported reason for choice of first provider in in-depth interviews. Hospital was sought when the disease was perceived to be severe (15/26). Most caretakers reported difficulties finding transport to go to the hospital.

Having reached a healthcare provider, caretakers could still withdraw the child from initiated care. This action was explained as due to a lack of money for continued hospital care and the anticipation of high costs of transporting a dead body.

*"People advised me that the child's health condition was beyond the hospital's capacity. The child became weaker every hour that was passing. I got scared that the child would die from hospital and yet I wouldn't have the means to transport the body, since my husband wasn't around. Luckily enough, there was a man who had visited us in the hospital who offered to carry us back home on his bicycle. So I requested the personnel to discharge us. The personnel however advised us that the child's blood should be examined, but I never had the money for blood testing and told the personnel that we would be back after getting some money. So we left the hospital for home." *36 year old mother of deceased 18 month old boy, less poor socioeconomic quintile.

### Barriers to receiving adequate treatment

Most caretakers reported caring at home for the sick child, normally starting treatment within minutes or hours after the recognition of illness. Paracetamol and chloroquine were the most commonly used drugs.

All but three children reached a healthcare provider outside the home. Caretakers were more content with nearby providers than the hospital. Private providers were appreciated for offering 24 hour-service, while both public and private providers were valued for making referrals when needed. The main complaints about the hospital concerned having to pay to get treated, long queues and rude staff. There also seemed to be a lack of triage with severely ill children not being attended to faster than the non-severely ill.

*"There is a lot of bureaucracy in getting medical care, for example, the child was in a bad health state but I was told to buy an exercise book and also get a patient number before the child could be attended to." *37 year old mother of deceased 7 month old girl, least poor socioeconomic quintile (exercise books are used for recording case notes in this setting).

Waiting times before being attended to were generally reported to be longer at the hospital compared to smaller facilities. Lack of drugs and blood were substantial problems at the hospital. Caretakers had frequently been advised to buy drugs, needles or infusion fluids, which contributed to further delays since caretakers had to spend time looking for money to buy the required equipment.

*"In the hospital, we stayed in a very long queue but even after enduring, the child was diagnosed and he was found to lack blood. Unfortunately, the child's blood group wasn't in stock and we were referred to Nalufenya children's hospital in Jinja. In Nalufenya hospital, we also stayed in a very long queue. After taking the blood samples, we were also told that the child's blood group wasn't in stock. The child was instead put on a water drip." *44 year old grandmother of deceased 7 month old boy, less poor socioeconomic quintile.

In some cases, health workers seemed to discharge or not admit patients who most likely needed (continued) hospital care.

*"We took the child to hospital and he was diagnosed and given treatment but we were told that admission was not necessary. So we went back home and were to return the following day. However, on a sad note, the child's health worsened that very night and yet we never had a way out and he passed away." *40 year old mother of deceased 5 month old boy, most poor socioeconomic quintile.

Some caretakers also gave reports of nonchalance among providers and rude reception.

*"I told *[the staff]*my child was very sick and I wanted to be registered. The man told me to bribe him before he could register me. He was just sitting there holding his pen. He told me he wanted 2000. I told him to just help me, the child is very sick. But he was very adamant." *34 year old mother of deceased 12 month old boy, poor socioeconomic quintile.

Of children with reported DRB (n = 16), all but one had been taken outside the home for care. Two were brought to private clinics only, while the remainder were brought to hospital only (n = 5), health centre only (n = 2) or both (n = 6). Three of these children had reportedly received antibiotics from the provider, while ten had received antimalarials and antipyretics only. Children with no reported DRB were treated at home for a median of three days compared to a median of one day for children with reported DRB, and were taken to private providers to a larger extent than children with DRB (RR 2.7, 1.0–7.0).

## Discussion

We describe a limited importance of traditional illness interpretations in the fatal outcome of febrile childhood illness. Prolonged intra-household decision processes or lack of access to cash and perceived poor quality of care at healthcare providers contributed to treatment failures. At another level, our results highlight resource constraints at different levels as the underlying barrier for adequate care of children with fatal acute febrile illness.

Three children reportedly received traditional care outside the home. While it has been suggested that traditional care is no longer a risk for delayed hospital care in Tanzania [27], the illness concept "lwenyanja" in this study led to traditional care rather than allopathic in late stages of illness. Even if not resulting in traditional care, local illness concepts may prompt inappropriate treatment as exemplified in this study by "enhonhi", which lead to prolonged home treatment and delayed hospital care. We believe that despite a limited importance overall, certain local illness concepts and traditional care still merit consideration in this setting.

Several children with reported difficult/rapid breathing were labeled as suffering from "omusudha" and were reportedly treated with antipyretics and/or antimalarials only. Only one caretaker suggested pneumonia as a cause of death, despite many children reportedly suffering from difficult or rapid breathing and even chest-indrawing. This indicates low awareness of pneumonia as a common childhood killer [7] and a need to improve caretakers' recognition and treatment of pneumonia.

Some women delayed to seek care since they first had to get money or approval from their husband. As in many other settings, women in Iganga/Mayuge carry the main responsibility for childcare but have limited access to financial resources [28]. Due to polygamy, a father is often head of several households, leaving his wives uncertain of where to find him. Since delays caused by gender-roles may be life-threatening [29], bringing effective diagnosis and free treatment for febrile illnesses closer to where women live may help mothers overcome financial barriers in care-seeking [30]. While it is known that several contextual and individual factors influence care seeking [31], further research is needed to understand how gender-roles interact with household socioeconomic status affecting health-seeking behavior [32].

Long waiting times, harshness from staff and lack of necessary drugs/equipment were major problems at the hospital according to caretakers. Health worker attitudes may influence decisions regarding how soon and where to treat the child [33], as well as caretakers' eventual compliance to treatment [34]. The extent of and reasons for health worker delays in attending to severely ill children and/or discharging them early needs further investigation and may require revision of hospital management protocols [35]. While the ability to provide adequate care may be limited due to resource constraints, effectiveness may be further reduced by poor adherence to hospital protocols and dysfunctional patient-practitioner relations. Quality of care could be improved through supervision and training of staff [36] and monitoring of quality of care [15].

Financial constraints at the individual level and resource constraints in the health system emerged as an underlying theme of all barriers to adequate care for the deceased children in this study. Out-of-pocket payments for illness can have serious economic consequences for already poor households [37], and fear of having to pay for transporting a dead body seemed to fuel requests for early discharge. Concentration of deaths in the poorest quintiles may reflect an inequity pattern with less-poor children receiving more appropriate care than the poorest [38]. Results indicate an urgent need to implement existing recommendations for the strengthening of health systems with limited resources [14] and for the improvement of health provision directed to the poor [39].

Due to the emotional difficulties associated with narrating the time preceding the death of a child, some in-depth interviews were less informative than others. Self-reporting may be affected by caretakers' memory and will to have done "the right thing". All children were less than two years old, perhaps due to their greater susceptibility to severe disease. Predictive values of Verbal Autopsies for determination of cause of death varies depending on local patterns of burden of disease [40]. We therefore abstain from presenting causes of death from VA interviews and use only symptoms to stratify the material. The long illness episodes reported by some caretakers may reflect the belief that one illness disappears and reappears over a long time-period [41], or an underlying HIV-infection. Discontent with providers may have been underreported due to worries it might have consequences on future possibilities to receive healthcare.

## Conclusion

Local illness concepts have an important, though limited, influence on the care-seeking for childhood febrile illness in this setting. While decision making on behalf of the caretakers and household lack of money contributed to delayed care-seeking, the majority of children were brought to, and reached, an allopathic health care provider during the illness leading to death. Improved management of children suffering from acute febrile illness by health care providers may thus contribute to a reduction of fatal outcomes. While some children may have required intensive high-cost care, the majority of caretakers revealed substantial deficiencies in provision of basic health care. Private and public health care providers at all levels should receive training, support, equipment and supplies to enable basic health care for children suffering from common illnesses.

## Competing interests

The authors declare that they have no competing interests.

## Authors' contributions

HH and SP designed the study protocol. HH and JK trained interviewers and participated in data collection. HH, GT and SP performed initial analysis of data and discussed analysis and results with all authors. HH wrote the first draft of the manuscript. All authors read and edited the manuscript.

## Pre-publication history

The pre-publication history for this paper can be accessed here:



## References

[B1] Jones G, Steketee RW, Black RE, Bhutta ZA, Morris SS (2003). How many child deaths can we prevent this year?. The Lancet.

[B2] Bojalil R, Kirkwood BR, Bobak M, Guiscafre H (2007). The relative contribution of case management and inadequate care-seeking behaviour to childhood deaths from diarrhoea and acute respiratory infections in Hidalgo, Mexico. Trop Med Int Health.

[B3] Reyes H, Perez-Cuevas R, Salmeron J, Tome P, Guiscafre H, Gutierrez G (1997). Infant mortality due to acute respiratory infections: the influence of primary care processes. Health Policy and Planning.

[B4] Task Force on Health Systems Research (2004). Informed choices for attaining the Millennium Development Goals: towards an international cooperative agenda for health-systems research. The Lancet.

[B5] Hill Z, Kendall C, Arthur P, Kirkwood B, Adjei E (2003). Recognizing childhood illnesses and their traditional explanations: exploring options for care-seeking interventions in the context of the IMCI strategy in rural Ghana. Tropical Medicine & International Health.

[B6] Pike KL (1954). Language in relation to a unified theory of the structure of human behaviour.

[B7] Hildenwall H, Rutebemberwa E, Nsabagasani X, Pariyo G, Tomson G, Peterson S (2007). Local illness concepts – Implications for management of childhood pneumonia in eastern Uganda. Acta Tropica.

[B8] Nsungwa-Sabiiti J, Kallander K, Nsabagasani X, Namusisi K, Pariyo G, Johansson A, Tomson G, Peterson S (2004). Local fever illness classifications: implications for home management of malaria strategies. Tropical Medicine & International Health.

[B9] Kallander K, Nsungwa-Sabiiti J, Balyeku A, Pariyo G, Tomson G, Peterson S (2005). Home and community management of acute respiratory infections in children in eight Ugandan districts. Annals of Tropical Paediatrics: International Child Health.

[B10] Kallander K, Nsungwa-Sabiiti J, Peterson S (2004). Symptom overlap for malaria and pneumonia – policy implications for home management strategies. Acta Tropica.

[B11] Peterson S, Nsungwa-Sabiiti J, Were W, Nsabagasani X, Magumba G, Nambooze J, Mukasa G (2004). Coping with paediatric referral – Ugandan parents' experience. The Lancet.

[B12] Nolan T, Angos P, Cunha AJLA, Muhe L, Qazi S, Simoes EAF, Tamburlini G, Weber M, Pierce NF (2001). Quality of hospital care for seriously ill children in less-developed countries. The Lancet.

[B13] English M, Esamai F, Wasunna A, Were F, Ogutu B, Wamae A, Snow RW, Peshu N (2004). Assessment of inpatient paediatric care in first referral level hospitals in 13 districts in Kenya. The Lancet.

[B14] Travis P, Bennett S, Haines A, Pang T, Bhutta Z, Hyder AA, Pielemeier NR, Mills A, Evans T (2004). Overcoming health-systems constraints to achieve the Millennium Development Goals. The Lancet.

[B15] Ntoburi S, Wagai J, Irimu G, English M (2008). Debating the quality and performance of health systems at a global level is not enough, national debates are essential for progress*. Tropical Medicine & International Health.

[B16] UNICEF/WHO (2006). Pneumonia the forgotten killer of children UNICEF/HQ05-0531. http://www.unicef.org/publications/files/Pneumonia_The_Forgotten_Killer_of_Children.pdf.

[B17] Kallander K, Hildenwall H, Waiswa P, Galiwango E, Peterson S, Pariyo G (2008). Delayed care seeking for fatal pneumonia in children under five in Uganda. Bulletin of the World Health Organization.

[B18] Uganda Bureau of Statistics Uganda Demographic and Health Survey 2000–2006.

[B19] Greene JC, Caracelli VJ, Graham WF (1989). Toward a Conceptual Framework for Mixed-Method Evaluation Designs. Educational Evaluation and Policy Analysis.

[B20] Indepht-Network Standard Verbal Autopsy. http://www.indepth-network.org/core_documents/VA_Template.zip.

[B21] Aguilar AM, Alvarado R, Cordero D, Kelly P, Zamora A, Salgado R (1998). Mortality Survey in Bolivia: The Final Report. Investigating and Identifying the Cause of Death for Children Under Five.

[B22] Setel PW, Whiting DR, Hemed Y, Chandramohan D, Wolfson LJ, Alberti KGMM, Lopez AD (2006). Validity of verbal autopsy procedures for determining cause of death in Tanzania. Tropical Medicine & International Health.

[B23] Filmer D, Pritchett LH (2001). Estimating wealth effects without expenditure data – or tears: an application to educational enrollments in states of India. Demography.

[B24] Graneheim UH, Lundman B (2004). Qualitative content analysis in nursing research: concepts, procedures and measures to achieve trustworthiness. Nurse Education Today.

[B25] Mayring P (2000). Qualitative Content Analysis. Forum Qualitative Sozialforschung/Forum: Qualitative Social Reserach.

[B26] Hynson JL, Aroni R, Bauld C, Sawyer SM (2006). Research with bereaved parents: a question of how not why. Palliative Medicine.

[B27] de Savigny D, Mayombana C, Mwageni E, Masanja H, Minhaj A, Mkilindi Y, Mbuya C, Kasale H, Reid G (2004). Care-seeking patterns for fatal malaria in Tanzania. Malaria Journal.

[B28] Orubuloye IO, Caldwell JC, Caldwell P, Bledsoe CH (1991). The impact of family and budget structure on health treatment in Nigeria. Health transition review.

[B29] Tolhurst R, Nyonator FK (2006). Looking within the household: gender roles and responses to malaria in Ghana. Transactions of the Royal Society of Tropical Medicine and Hygiene.

[B30] Tolhurst R, Amekudzi YP, Nyonator FK, Bertel Squire S, Theobald S (2008). "He will ask why the child gets sick so often": The gendered dynamics of intra-household bargaining over healthcare for children with fever in the Volta Region of Ghana. Social Science & Medicine.

[B31] Kamat VR (2008). Dying under the Bird's Shadow. Medical Anthropology Quarterly.

[B32] Ajayi I, Browne E, Garshong B, Bateganya F, Yusuf B, Agyei-Baffour P, Doamekpor L, Balyeku A, Munguti K, Cousens S (2008). Feasibility and acceptability of artemisinin-based combination therapy for the home management of malaria in four African sites. Malaria Journal.

[B33] Gross GJ, Howard M (2001). Mothers' Decision-Making Processes Regarding Health Care for Their Children. Public Health Nursing.

[B34] D'Souza RM (2003). Role of Health-Seeking Behavior in Child mortality in the slums of Karachi, Pakistan. Journal of Biosocial Science.

[B35] Sodemann M, Jakobsen M, Molbak K, Alvarenga I, Aaby P (1997). High mortality despite good care-seeking: a community study of childhood death in Guinea-Bissau. Bulletin of the World Health Organization.

[B36] Rowe AK, de Savigny D, Lanata CF, Victora CG (2005). How can we achieve and maintain high-quality performance of health workers in low-resource settings?. The Lancet.

[B37] McIntyre D, Thiede M, Dahlgren G, Whitehead M (2006). What are the economic consequences for households of illness and of paying for health care in low- and middle-income country contexts?. Social Science & Medicine.

[B38] Schellenberg JA, Victora CG, Mushi A, de Savigny D, Schellenberg D, Mshinda H, Bryce J (2003). Inequities among the very poor: health care for children in rural southern Tanzania. The Lancet.

[B39] Gwatkin DR, Bhuiya A, Victora CG (2004). Making health systems more equitable. The Lancet.

[B40] Korenromp EL, Williams BG, Gouws E, Dye C, Snow RW (2003). Measurements of trends in childhood malaria mortality in Africa: an assessment of progress toward targets based on verbal autopsy. The Lancet infectious diseases.

[B41] Hausmann Muela S, Muela Ribera J, Mushi AK, Tanner M (2002). Medical syncretism with reference to malaria in a Tanzanian community. Social Science & Medicine.

